# Deeply Diverged but Morphologically Conserved Lineages in Tornier's Cat Snake (*Crotaphopeltis tornieri*) of the Eastern Arc Mountains

**DOI:** 10.1002/ece3.70452

**Published:** 2025-02-25

**Authors:** Tejs L. Nielsen, Sofie Holdflod Nielsen, Maria Novosolov, Peter Gravlund, Morten E. Allentoft

**Affiliations:** ^1^ Section for GeoGenetics Globe Institute, University of Copenhagen Copenhagen Denmark; ^2^ Natural History Museum of Denmark, University of Copenhagen Copenhagen Denmark; ^3^ Trace and Environmental DNA (TrEnD) Laboratory School of Molecular and Life Sciences, Curtin University Perth Australia

**Keywords:** Colubrids, Eastern Arc Mountains, mitochondria, morphology, population genetics

## Abstract

The Eastern Arc Mountain (EAM) forests in Tanzania have remarkably high endemism. Closely‐related forest‐adapted species are found isolated on different “sky islands” testifying to allopatry as a major driver for speciation in this region. However, some species defy this pattern. Tornier's cat snake (*Crotaphopeltis tornieri*) occupies most of the isolated mountain rainforest, despite presumably not being able to move across the arid savannah landscape that separates them. To test contrasting hypotheses of recent dispersal vs morphological conservatism we examined scale characters of 218 *C. tornieri* individuals and sequenced 80 full mitochondrial genomes covering populations from eight mountain blocks across the EAM and Southern Highlands of Tanzania (SHT). The morphological examination revealed no differentiation between populations except the Usambara Mountain populations showing significant differences in some scale characters. This was in stark contrast to the genetic analyses showing very high divergence between mountain populations. On average the mitochondrial genome showed > 12% genetic differentiation with cytB and COI showing interpopulation distances of up to 28.5% and 15.1%, respectively. Both Bayesian coalescent and maximum‐likelihood based phylogenies, uncovered a highly distinct clade structure in *C. tornieri* defined by the mountains. Divergence times were estimated at c. 21 million years for the split between the EAM and SHT populations and 5.4–1.4 millions years for population splits within EAM. Our results point towards old isolation events but with a highly conserved morphology resulting in just one recognized species. By including presumed outgroups of *C. degeni* and *C. hotamboeia* in the phylogeny we found *C. tornieri* to be paraphyletic. These results have implications for understanding evolution in the EAM and warrant a revision of the number of species in this genus.

## Graphical abstract

Populations of the forest‐adapted snake species *Crotaphopeltis tornieri* are found across the Eastern Arc Mountains in Tanzania By analysing mitochondrial genomes from 80 individuals distributed across six of the mountains along with the Southern Highlands of Tanzania, we found very high genetic variation between the populations (> 12%) indicating millions of years of separation In contrast, morphological analyses revealed little to none variation, indicating possible cryptic speciation.

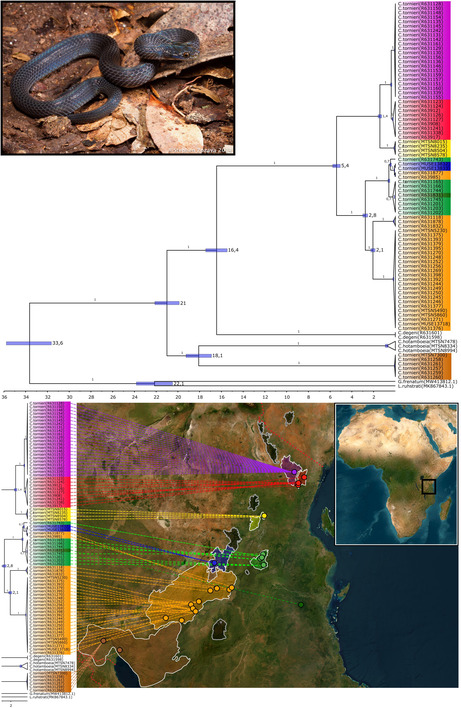

## 1 Introduction

**FIGURE 1:**
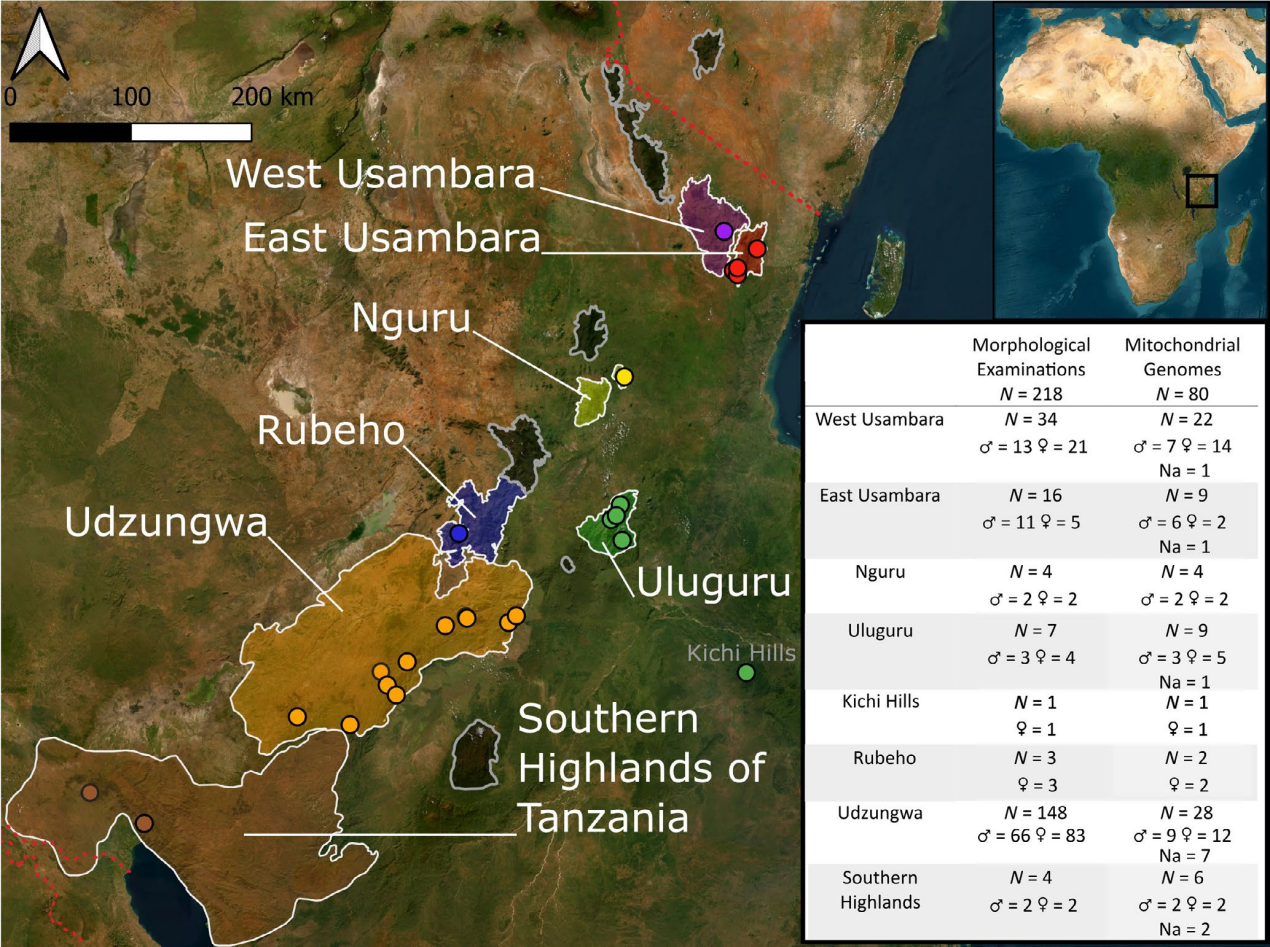
Showing the different Eastern Arc Mountains (polygons) and the sampling locations for the *Crotaphopeltis tornieri* mountain populations (dots), along with the number (*N*) of morphologically examined specimens and number of mitochondrial genomes. The number of each sex is also shown, and in cases where the sex is unknown is shown as ‘Na’. Greyed‐out mountains were not included in this study.

The Eastern Arc Mountains (EAM) is a chain of segregated crystalline mountain blocks in Eastern Africa, which uplifted due to faulting in the earth's crust. They extend from the Taita Hills in southern Kenya to the Udzungwa Mountains in south‐central Tanzania (Figure 1) (Burgess, Fjeldså, and Botterweg 1998; Burgess et al. 2007; Newmark 2002; Wasser and Lovett 1993). Reaching an elevation of 2635 m a.s.l. (Burgess, Fjeldså, and Botterweg 1998; Burgess et al. 2007), the mountains act as a blockade against the oceanic winds from the Indian ocean and are thus affecting the climate in the otherwise savannah dominated landscape of Tanzania (Rodgers 1998; Wasser and Lovett 1993). This ensures sufficient precipitation to support dense montane rainforest, today distributed as fragmented patches of this habitat (Rodgers 1998; Wasser and Lovett 1993; Meadows and Chase 2007; Prell et al. 1980). The EAM and the coastal forests of the area have the highest recorded density of endemic species with more than 80 endemic species of vertebrates and plants recorded per 100 km^2^ (Myers et al. 2000). More than 50% of the EAM endemic vertebrate species are endemic to single mountain blocks (Rovero et al. 2014), and this number has been found to be as high as 90% for invertebrate species (Burgess et al. 2007; Scharff 1993).

Understanding the underlying mechanisms responsible for such high endemism holds an important key to understand the formation and distribution of biodiversity in montane ecosystems worldwide (Rahbek et al. 2019). Three main mechanisms have been suggested to explain the high endemism found in the EAM: (i) The ‘species pump hypothesis’ suggests that forest fragmentation during dry periods, and forest expansion and reconnection during humid periods, acts as a ‘speciation pump’ generating new biodiversity at an elevated rate (Couvreur et al. 2008; Dimitrov, Nogues‐Bravo, and Scharff 2012; Gravlund 2002; Lovett 1993b; Measey and Tolley 2011; Rodgers 1998). This process of forest fragmentation has a profound effect on the distribution of regional flora and fauna resulting in numerous allopatric speciation events (Dimitrov, Nogues‐Bravo, and Scharff 2012; Lawson 2010; Measey and Tolley 2011). Phylogenetic studies have pointed towards vicariance as a major driver of the allopatric speciation events in the EAM, showing multiple examples of distinct but closely related forest‐restricted species distributed across the different mountain blocks (Couvreur et al. 2008; Matthee, Tilbury, and Townsend 2004; Menegon et al. 2014; Tolley et al. 2011; Voelker, Outlaw, and Bowie 2010). (ii) The ‘refuge hypothesis’ suggests that the remarkably high biodiversity in the EAM is a result of extreme environmental stability, where the isolated montane forests acts as relicts, potentially dating back to the extensive prehistoric Pan African Rainforest that covered the whole region 30 million years ago (Howell 1993; Lovett 1993a, 1993b; Wasser and Lovett 1993). This tremendous buffering capacity of the system, where the forests do not disappear during dry periods but rather retract to higher elevations, have protected lineages during severe and prolonged climatic events that caused global extinctions elsewhere (Fjeldså and Bowie 2008; Lovett 2005). As a result, the forests today boast a plethora of old evolutionary lineages within numerous taxonomic groups (Burgess, Fjeldså, and Botterweg 1998; Tolley et al. 2011). And (iii) The ‘elevated in situ speciation rate hypothesis’ suggests that local gene flow breaks as a result of physical barriers (e.g., rivers, valleys, forest gaps, rocky escarpments resulting in a more recent lineage diversifications in the montane forests or along the foothills; Burgess et al. 2007; Fjeldsaå and Lovett 1997). By showing evidence of relatively young lineages present in the forests, several molecular studies have found supporting evidence for this mechanism in the EAM (Beresford, Fjeldså, and Kiure 2004; Bowie and Fjeldså 2005, Kahindo, Bowie, and Bates 2007, Dimitrov, Nogues‐Bravo, and Scharff 2012; Loader et al. 2004; Lindqvist and Albert 2001; Roy 1997; Scharff 1993).

However, certain species in the EAM seem to defy the typical vicariance patterns (Ceccarelli et al. 2014; Menegon et al. 2014; Burgess et al. 2007; Blackburn and Measey 2009). Examples of species that are distributed across several of the mountain blocks despite being poor dispersers, and restricted to the mountain forests, are not uncommon. Among the herpetofauna such examples include the chameleon species *Trioceros werneri* found on Nguru, Udzungwa, Ukaguru and Uluguru (Ceccarelli et al. 2014), the bush viper *Atheris ceratophora* (Menegon et al. 2014) found in the Usambara and Udzungwa, the Caecilian *S* *colecomorphus vittatus* (Burgess et al. 2007), and the frog *Arthroleptidae xenodactyloides*, (Blackburn and Measey 2009). A recently published example among arthropods includes the millipedes *Tropostreptus hamatus and T. sigmatospinus* (Enghoff 2017; Nielsen et al. 2022). The cross‐mountain distribution of these putatively poor dispersers is an Eastern Arc enigma that is not well accounted for in the currently proposed hypotheses and thus it deserves attention when studying the evolutionary mechanisms in the EAM.

One such enigmatic example among the highly diverse herpetofauna of the EAM is the snake species *Crotaphopeltis tornieri* commonly known as ‘Tornier's Cat Snake’ or ‘Werner's Water Snake’ (Werner 1908). *Crotaphopeltis tornieri* is a 30‐ to 50‐cm snake with a cylindrical body, broad head, and a short tail (10% of total length) (Spawls et al. 2018). It has red or orange‐red eyes of moderate size with black vertical pupils, indicating nocturnal activity similar to other *Crotaphopeltis* species (Rasmussen 1993). The species is nearly endemic to the EAM and has a distribution across different mountain blocks, despite being restricted to montane forests and presumably unable to migrate between them (Spawls et al. 2018). The species occupies at least six mountain blocks within the EAM, ranging from West Usambara in the North to Udzungwa Scarp in the South, along with the Southern Highlands of Tanzania (SHT) and the Misuku mountains, Malawi, further southwest (Rasmussen 1993; Spawls et al. 2018). There are three plausible scenarios that could explain its current distribution: (i) Long range dispersal between the mountains, maintaining gene flow. This is theoretically possible for *C. tornieri* as it has been collected at elevation down to 600 m a.s.l. at the Amani Nature Reserve, East Usambara (R631742, ZMUC collection, species identification confirmed morphologically and genetically, unpublished data). Moreover, there are several rare recordings of specimens collected from non‐montane habitats (e.g., Vikindu forest reserve—< 100 m a.s.l., Magombera forest reserve—300 m a.s.l.) and even near coastal cities (e.g., Lindi, Tanga; Rasmussen 1993; albeit these could represent species misidentifications or mislabelling of locality). However, the idea of at least six independent long‐range dispersal events across the savannah landscape in the very recent evolutionary history of this species seems implausible; (ii) A recent vicariance event, resulting in populations being isolated on each mountain block but with insufficient time to evolve into separate species. However, several studies have indicated that the mountain forests have been isolated for millions of years, not supporting a theory of recent vicariance events (Ceccarelli et al. 2014; Menegon et al. 2014; Tolley et al. 2011); and (iii) High morphological conservatism in a lineage with several distinct species (Austin 1995; Moen, Irschick, and Wiens 2013), possibly linked to phylogenetic niche conservatism (Wiens 2004). Previous molecular studies of *C. tornieri* based on one to two mitochondrial genes have favoured this scenario owing to high genetic divergence between the separated mountain populations and indications of paraphyly (Gravlund 2002; Engelbrecht et al. 2020).

In realising the wider implications for understanding Eastern Arc evolution, we conducted phylogenetic analyses based on 80 full mitochondrial genomes of *C. tornieri* museum specimens covering six EAM blocks along with Mount Rungwe and the Madehani Forest Reserve in the SHT. We also sequenced full mitogenomes from species *C. hotamboeia*, and *C. degeni* as putative outgroups to *C. tornieri*. Moreover, in assessing the theory of morphological conservatism we conducted a morphological examination of scale characters on 218 *C. tornieri* individuals, supplementing previous efforts (Rasmussen 1993) with more individuals and more populations. The findings of this study improves our understanding of the evolutionary processes in the EAM, particularly in relation to species displaying highly disjunct distributions like *C. tornieri*.

## 2 Material and Methods

### 2.1 Morphological Analyses

**FIGURE 2:**
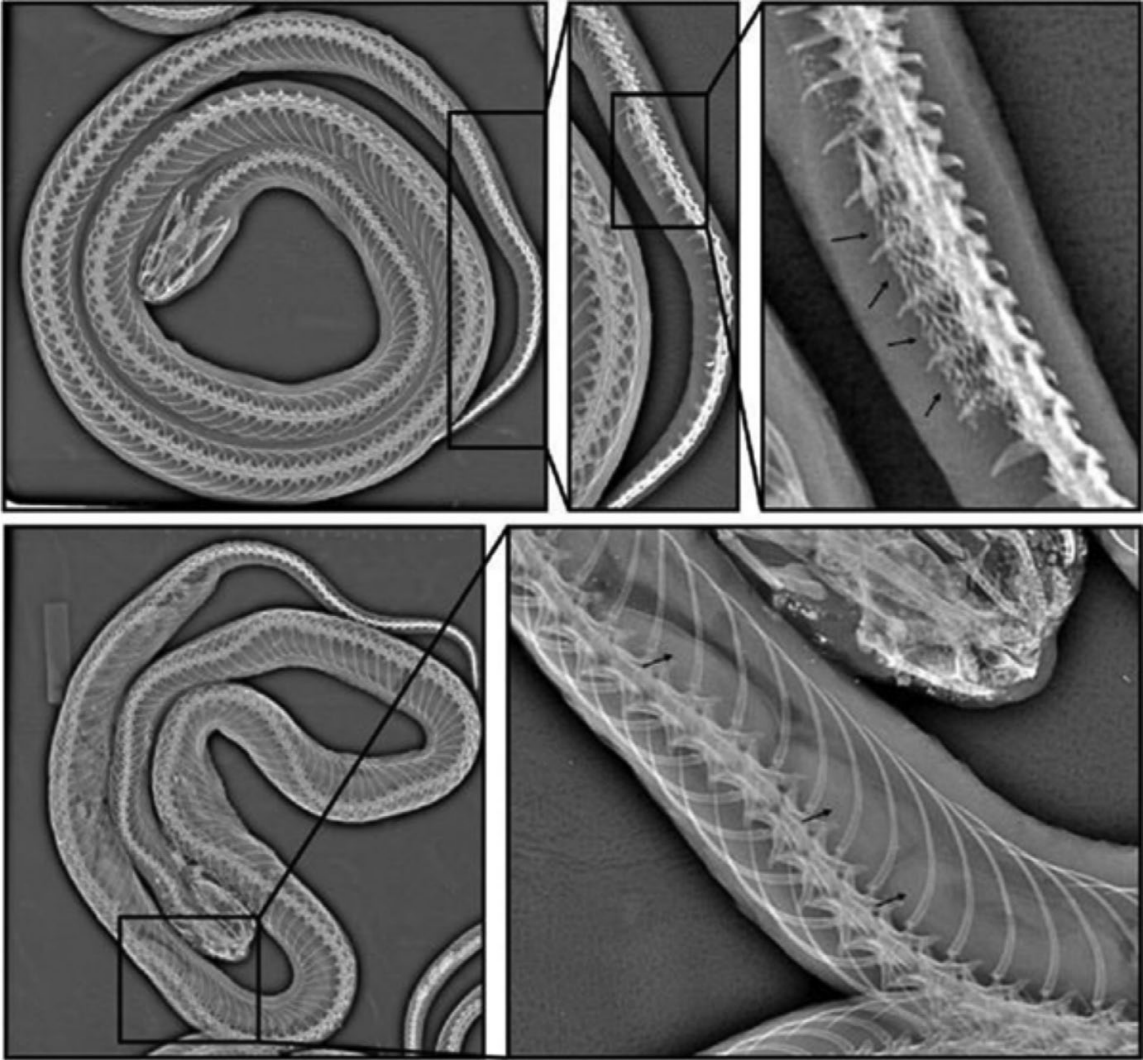
Top shows a radiographic image of a male specimen of *Crotaphopeltis tornieri*, visualising the full body, focus on the tail, and calcified ‘spines’ on the hemipenis (arrows). Bottom shows a radiographic image of a female specimen of *C. tornieri* with eggs, visualising the full body and eggs (arrows).

A total of 235 ethanol‐preserved museum specimens of *C. tornieri* from the Museo delle Scienze Trento, Italy (*n* = 15), and the Natural History Museum of Denmark (*n* = 220) were initially morphologically examined for this study but only 218 qualified for detailed morphological analyses (see explanation below). We included specimens representing seven blocks in the EAM (Figure 1)—including SHT which does not share the same geological history as the EAM (Ring 2014; Rodgers 1998). The specimens were sexed, with males determined by the presence of hemipenes and females by the presence of eggs and/or the absence of hemipenes. In cases where sexing was difficult, we determined this using *x*‐ray scans (Figure 2; Gnudi et al. 2009). A total of 13 meristic traits were recorded: ventral scale count (VSC); anterior ventral scale count (AVS); number of scale rows at midbody (MSR); subcaudal scale count (SCS); the upper labial scale count (LSC), including the upper labials bordering the eye (LOC); infralabial scale count (ILC) including the infralabial scales in contact with the anterior chin shield (ICS); the temporal scale counts (TSC); preocular scale count (PRO) and postocular scale count (POO); the relative heart position (HP) in relation to the VSC; along with presence/absence of the keeled dorsal scales (KVS) were recorded (Table S1). The scale counts traits were primarily examined following the methods used by Rasmussen (1993), with following exceptions: MSR only counted at mid body, SCS only counted on the left side, VSC and AVS counted following the method of Dowling (1951). The exact method for counting the scale count traits, along with abbreviations are described in Table S1. For traits variating between each side of the specimen, only the left side was used in the morphological analyses.

The software PAST.3 (Hammer and Harper 2001) was used for all morphological statistical analyses—Principal component analysis (PCA), canonical variates analysis (CVA), boxplot and Mann–Whitney pairwise test (Mann and Whitney 1947). Mann–Whitney pairwise tests were chosen because of varying sample sizes and performed on ‘high‐range traits’, that is, VSC, SCS and HP. The Mann–Whitney pairwise test was conducted using all individuals with the specific trait recorded, including individuals which did not have all traits recorded. The number of individuals for each analysis can be seen in Table 1. For the clustering analyses, only individuals with all morphological traits recorded were included in the PCA and CVA, resulting in 181 individuals (Figure 1). All data for PCA and CVA were standardised for each trait by dividing each data point by the corresponding overall standard deviation (SD). Morphological analyses were performed on female and male specimens separately due to the sexual dimorphism in snout‐vent length and thus associated scale counts traits (King 1989; Lee, Thompson, and Mulcahy 2016; Rasmussen 1993). Separating the sexes resulted in small sample sizes for individual mountains, thus levels of significance should be interpreted with caution. Due to lack of variation in KVS it was excluded from PCA and CVA analyses for the males.

**TABLE 1:** Results of Mann–Whitney pairwise test (Mann and Whitney 1947), testing for significant morphological differences in ventral scale count (VSC), subcaudal scale count (SCS), and relative heart position (HP), between populations in. Results from females are shown above the diagonal and results from males below the diagonal. Numbers in brackets indicate the number of individuals per population in the corresponding analyses, males vertically and females horizontally. ‘*’ symbolises significant differences at *p* < 0.05 and ‘***’ symbolises significant differences at *p* < 0.01.

Males\females	VSC						
	West Usambara (21)	East Usambara (4)	Nguru (2)	Rubeho (3)	Uluguru (4)	Udzungwa (77)	SHT (2)
West Usambara (13)		0.003	0.621	0.204	0.219	2.19e^−6^***	0.784
East Usambara (10)	6045e^−05^***		0.159	0.052	0.081	8477e^−4^***	0.105
Nguru (2)	0.864	0.038*		0.554	0.817	0.060	1000
Rubeho (0)	—	—	—		0.112	0.638	0.554
Uluguru (2)	0.173	0.064	0.699	—		0.005***	0.247
Udzungwa (60)	3914e^−04^***	4966e^−07^***	0.082	—	0.034*		0.100
SHT (2)	0.733	0.038*	0.699	—	0.245	0.149	
	**SCS**						
	**West Usambara (21)**	**East Usambara (5)**	**Nguru (2)**	**Rubeho (3)**	**Uluguru (4)**	**Udzungwa (80)**	**SHT (2)**
West Usambara (13)		0.002***	0.617	0.021*	0.004***	6929e^−05^***	1000
East Usambara (11)	0.008***		0.076	0.749	0.381	0.606	0.076
Nguru (2)	0.087	0.369		0.224	0.105	0.210	1000
Rubeho (0)	—	—	—		0.359	0.893	0.224
Uluguru (2)	0.032*	0.046*	0.245	—		0.118	0.105
Udzungwa (62)	0.001***	0.200	0.938	—	0.062		0.113
SHT (2)	0.146	1000	0.414	—	0.245	0.426	
	**HP**						
	**West Usambara (21)**	**East Usambara (3)**	**Nguru (2)**	**Rubeho (3)**	**Uluguru (4)**	**Udzungwa (76)**	**SHT (2)**
West Usambara (11)		0.541	0.957	0.541	0.250	0.507	0.043
East Usambara (10)	0.072		0.773	1000	0.860	0.608	0.773
Nguru (2)	0.767	0.914		0.773	0.488	0.884	0.245
Rubeho (0)	—	—	—		0.860	0.519	0.773
Uluguru (2)	0.622	0.451	0.699	—		0.448	0.247
Udzungwa (62)	0.682	0.007***	0.497	—	0.313		0.022*
SHT (2)	0.374	0.914	0.699	—	0.699	0.155	

### 2.2 DNA Sampling

It has been shown that snake tissues (muscle/skin, bone and liver) preserved in ethanol can retain a relatively high endogenous DNA content for decades and even centuries (Zacho et al. 2021). Liver tissue especially tends to produce high DNA concentrations (Zacho et al. 2021), thus we favoured liver tissue for the molecular component of this study. Liver samples were carefully removed from museum specimens by making lateral incisions between two ventral scales resulting in minimal external damage to museum specimens and the samples were stored at −18°C until DNA extraction. 85 out of 138 DNA‐libraries (Table S2) yielded sufficient quality for downstream analyses including 80 samples of *C. tornieri* (Figure 1) two samples of *C. degeni*, and three samples of *C. hotamboeia*. The age of the museum samples varied from 1978 to 2014. Samples of the remaining *Crotaphopeltis* species (*C. barotseensis*, *C. braestrupii* and *C. hippocrepis*) did not produce sufficient data quality for mitogenome assembly, and were therefore excluded from molecular analyses.

### 2.3 DNA Extraction

DNA was extracted using a Qiagen DNeasy 96 Blood and Tissue kit and the Kingfisher Cell and Tissue kit for the Duo Prime system, following the protocol provided by the manufacturer. The amount of starting material varied, but in most cases 10–50 mg liver tissue was removed. Samples that were poorly dissolved after 24 h of incubation (possibly because of formalin exposure), received an additional 40 μL proteinase‐K and an extra 24‐h incubation, before being extracted. One negative control‐sample was included for each extraction‐batch, using double distilled H_2_O (ddH_2_O). Because of expected varying degree of DNA preservation due to age difference (storage time) between the specimens, the extracted DNA was fragmented to an average length of 550 bp using a Covaris M220 Focused‐ultrasonicator (Covaris, Woburn, MA, USA) before being built into double‐stranded DNA libraries using NEBNext DNA Sample Prep Master Mix (New England Biolabs, Ipswich, MA, USA), and Illumina‐specific adaptors (Illumina, San Diego, CA, USA) following the protocol of Meyer and Kircher (2010) with some modifications (see Allentoft, Rasmussen, and Kristensen 2018). qPCR (Roche LightCycler 480) was used to estimate the number of optimal PCR cycles in library amplification. Negative control samples were included in all qPCR runs. The amplified libraries were examined using an Agilent 2200 Tapestation, measuring the DNA concentration and the DNA fragment lengths. After quantification, the libraries were pooled with equimolar concentration and finally ‘shotgun’ sequenced using 150 bp PE (paired‐end) chemistry on a combination of an Illumina Hiseq4000 V2 flowcell (375 m reads) and NovaSeq 6000 SPXP (350 m reads per lane) and SPXP4 (2.5b reads per lane) flowcells at the GeoGenetics sequences core (University of Copenhagen).

### 2.4 *De‐Novo* Assembly of Mitochondrial Genomes

The reads were base‐called using CASAVA 1.8.2 and de‐multiplexed with a full match requirement of the unique 2 × 6 nucleotide‐indices used. The raw reads were trimmed for adapters using AdaptorRemoval v.2.2.2 (Schubert, Lindgreen, and Orlando 2016) and reads shorter than 30 bp were discarded. Being paired‐end data, forward and reverse reads with 15 or more bp overlapping were collapsed. The iterative mapping algorithm implemented in MITObim (Hahn, Bachmann, and Chevreux 2013) was used to reconstruct the *de novo* reference mitochondrial genomes of the different *Crotaphopeltis* species. Using a singular reference for *C. tornieri* proved insufficient, with only conserved regions mapping successfully. Because of large genetic distances, it was necessary to run MITObim separately for each species and population to obtain usable reference sequences in the downstream mapping. For each reference reconstruction, the resulting sequence was confirmed in several MITObim runs based on different settings and using the following published colubrid mitogenomes as initial sequence‐bait: *Lycodon rufozonatum* (KJ179950.1), *Euprepiophis perlacea* (NC_024546.1), *Hebius vibakari ruthveni* (KP684155.1) and *Thermophis zhaoermii* (GQ166168.1). The resulting assembled *Crotaphopeltis* mitochondrial genomes, representing each species and population, were used as references in subsequent mapping of the shotgun data from all specimens. This was done with BWA v.0.7.15 (Li et al. 2009) using standard settings. The resulting bam files for all specimens were imported and visually inspected in Geneious Prime v.2019.2.1. (Kearse et al. 2012) to check that mapping coverage was homogeneous across the mitogenome, despite having sample‐specific variants compared to the respective reference. For each individual, a consensus sequence was then constructed by only including sites with read coverage > 5 (otherwise the position would remain undetermined) and with a conservative threshold of 90% agreement between reads for each site. Undetermined sites (< 90% agreement) were given IUPAC ambiguity codes.

High coverage mitogenomes representing individuals of *C. tornieri* (MTSN8578); *C. degeni* (R631601); *C. hotamboeia* (MTSN8334), were selected for annotation using MITOS with default settings ‘vertebrates’ selected (Bernt et al. 2013). Annotations were adjusted by visual inspection by comparison with the mitogenomes annotated through MITOS, and manually edited to ensure that each coding sequence began with a start codon ‘Met’ and ended at the first occurring stop codon. Each sequence was subsequently inspected in Geneious Prime v.2019.2.1 to check that mapping coverage was homogeneous across the mitogenome, despite obviously having sample‐specific variants compared to the respective reference.

### 2.5 Phylogenetic Analysis

The sequences were aligned using ‘clustal omega multiple align’ in Geneious Prime 2023.0.1 (Kearse et al. 2012), allowing the software to adjust the settings according to the input data (default setting). Two concatenated alignments were conducted for the mitogenomes: One with all coding sequences (CDS) and another with ribosomal RNA (rRNA) sequences. Using the three annotated mitogenomes as alignment references, individual CDS genes and rRNA sequences were identified in all individual mitogenomes and subsequently concatenated in the original order of the mitogenome, resulting in two alignments of 11,483 and 2481 bp respectively. The CDS and rRNA alignments were further concatenated into a final alignment with a total of 13,964 bp. The mitogenomes of colubrid species *Gonyosoma frenatum* (GenBank: MW413812.1) and *Lycodon ruhstrati* (GenBank: MK867843.1) were selected as outgroup to all *Crotaphopeltis* species. To build a phylogenetic tree, we used BEAST 2 v2.6.7 (Bouckaert et al. 2019) which uses a Bayesian Markov Chain Monte Carlo (MCMC) algorithm and a coalescent‐based framework. Input XML‐files were constructed in BEAUti 2 v2.6.7. The two concatenated alignments were linked using the linked clock and linked tree function. The substitution model and value of the shape and proportion of invariance were selected based on the best fit suggested by Jmodeltest v2.1.10 (Darriba et al. 2012; Guindon and Gascuel 2003), and subsequently adjusted to ensure higher effective sample sizes (ESS). For the CDS alignment, the substitution model General Time Reversible (GTR) was used, with a gamma category count of 6, gamma shape of 1.575 and proportion of invariance of 0.457. For the rRNA alignment, the TIM2 substitution model was used, with a gamma category count of 4, a gamma shape of 0.1 and proportion of invariance of 0.1. All parameters were allowed to be estimated by the software during the analyses under normal distribution. A strict clock rate of 7.32^E−9^ (substitutions per site per year), were selected based on the average mitogenome substitution rate for colubrids (Eo and DeWoody 2010). The phylogenetic analyses were conducted with a chain length of 100 million trees with every 1000^th^ tree logged. The log file was inspected using Tracer v.1.7.2 (Rambaut et al. 2018), showing all effective sample sizes (ESS) of parameters to be sufficient quality, for example, posterior ESS = 14,088 and likelihood ESS = 12,761, prior ESS = 8374. A maximum clade credibility tree was produced using TreeAnnotator v.2.6.7, with a burn‐in of 10%. The final tree was inspected and edited in Figtree v. 1.4.4 (http://tree.bio.ed.ac.uk/software/figtree/).

We built an additional phylogenetic tree using RAxML‐NG v. 0.8.1 Beta (Kozlov et al. 2019), which uses a maximum‐likelihood phylogenetic approach. The analysis was conducted using standard settings with the GTR substitution model, gamma distribution, proportion of invariable sites and 1000 bootstrap replicates. Furthermore, we conducted a haplotype network using a minimum spanning network in Popart (Bandelt, Forster, and Röhl 1999), only including *Crotaphopeltis* species in the alignment. The RAxML phylogenetic tree and the haplotype network are included as Figures S1–S3.

### 2.6 Pairwise Distance Analyses

Estimates of evolutionary divergence over sequence pairs between each specimen and between groups (populations and species) were made using MEGA 11 (Tamura, Stecher, and Kumar 2021). The rRNA and CDS alignments were concatenated for this analysis, using the concatenate function in Geneious Prime. The *p*‐distance model was selected with standard error estimates obtained by a bootstrap procedure of 10,000 replications. Nucleotides were chosen as substitution type, including transitions and transversions, using uniform rates. A total of 13,964 sites were used in this analysis and all ambiguous positions were removed for each sequence pair (pairwise deletion option). Codon positions included were 1st + 2nd + 3rd + Noncoding. All ambiguous positions were removed for each sequence pair (pairwise deletion option).

Associations between the genetic distance and geographical distance were analysed using the Mantel test with 100,000 permutations (Mantel 1967; Sokal 1979) in R V. 4.1.1 (R Core Team 2021). The analysis was only done for *C. tornieri* populations with the geographical input being the centre of the sampling locations for each population.

## 3 Results

### 3.1 Morphological Data

**FIGURE 3:**
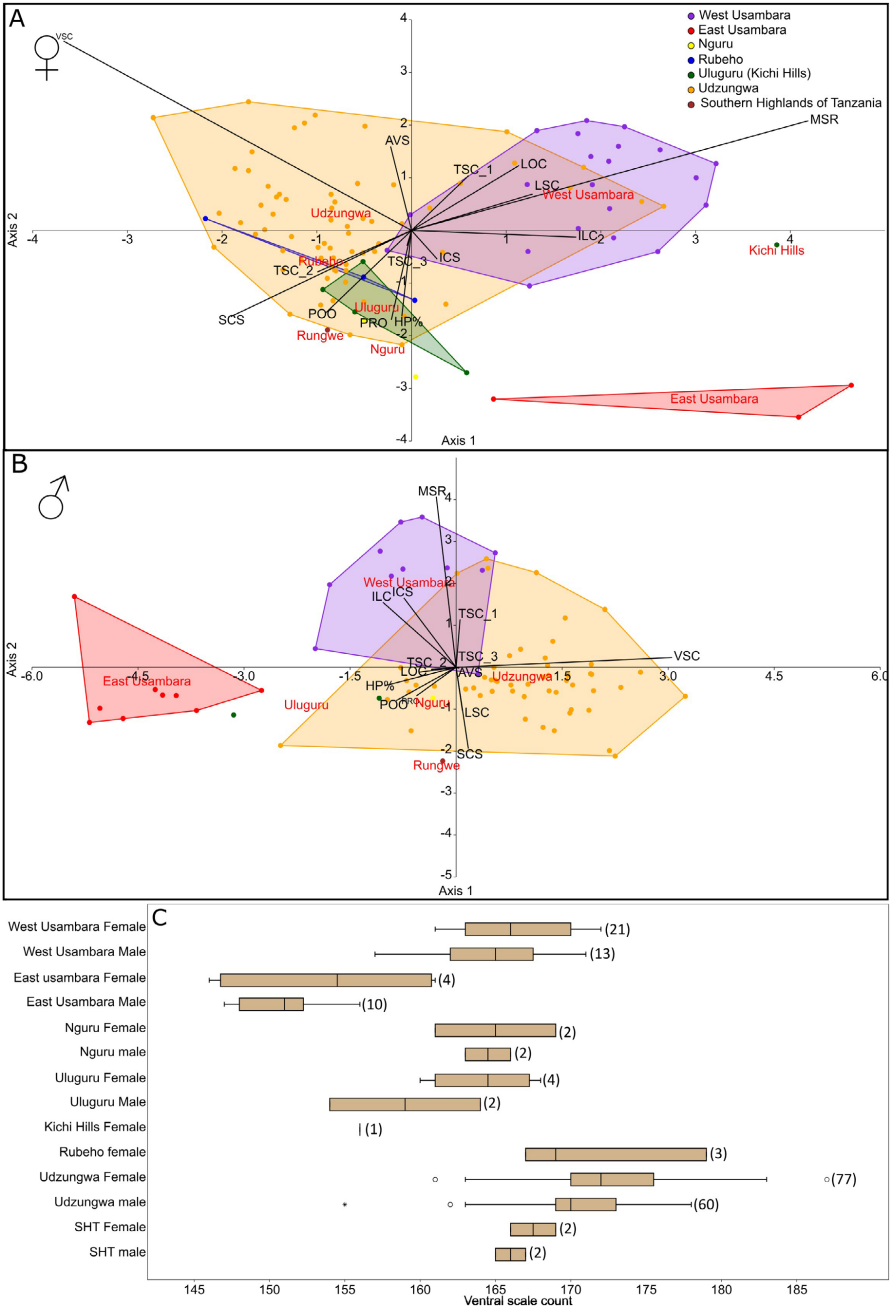
CVA‐plots of morphological data of 103 females (A) and 78 males (B) including all traits examined besides ‘KVS’. (A) The Jack‐knife analysis showed 52% agreement with the identified species with axis 1 and 2 explaining 53% and 23% of the variance. (B) The Jack‐knife analysis showed 72% agreement with the identified species with axis 1 and 2 explaining 63% and 18% of the variance. See Section 2 for bi‐plot abbreviations. (C) Boxplots of the variance in VSC for each sex divided per population, numbers of specimens are shown in brackets.

We were able to record all 13 morphological traits along with sex and location for 181 specimens (103 females and 78 males). Certain scale patterns proved common: LSC, 8 (3–5), LOC shown in brackets, in 82% of all individuals ILC, 10 (5), ICS shown in brackets, in 67% of all individuals; and TSC, 1 + 2 in 60% of all individuals. This is in agreement with the most common patterns found by Rasmussen (1993): LSC, 8 (3–5) (60%); ILC, 10 (5) (71%); and TSC, 1 + 2 (71%) respectively (Table S1). The chance of observing patterns deviating from the ‘common’ ones was strongly correlated with sample size, indicating that such deviating patterns occurred due to infrequent abnormalities rather than being population specific. Our morphological comparison of ‘high range’ traits in *C. tornieri* showed very little variation between populations (Table 1) albeit with a few exceptions outlined here: The East Usambara population proved significantly or close to significantly different in the VSC character compared to all other examined populations using the Mann–Whitney pairwise test (Table 1, Figure 3). The VSC in the males of the East Usambara population was significantly lower (*p* < 4.966e−07 to *p* = 0.038) compared to the other populations, except for the males from Uluguru (*p* = 0.64). Likewise, the East Usambara female population had significantly lower VSC compared to the females of Udzungwa (*p* = 8.477e−4) and West Usambara (*p* = 0.003) and a tendency to showing a difference to the remaining male populations (*p* = 0.052–159). The Mann–Whitney pairwise test of VSC also showed that the Udzungwa population was significantly different from both Usambara populations and the Uluguru population for both sexes (Table 1). The SCS counts revealed the West Usambara population to be significantly different to most populations for both sexes (*p* = 6.929e−05–0.032), with the exception of the Nguru and SHT populations. The relative HP did not show a clear trend, only showing differences between SHT and West Usambara and Udzungwa for the females and East Usambara and Udzungwa for the males (Table 1). We note that the two insignificant results were based on comparisons with a low number of individuals from only two specimens from Nguru, Rubeho, and Uluguru and SHT respectively.

Though not included in the Mann–Whitney pairwise test, the West Usambara population was found to be different in MSR, with a count of primarily 19 compared to all other *C. tornieri* populations primarily having 17, including the SHT population (Table S1). The KVS character and the remaining head scale characters (Table S1), did not reveal any apparent differences.

None of the populations differentiated significantly in the cluster analyses (PCA and CVA) for either sex when examining all 13 traits together and using 95% confidence limits (Figure 3, Figure S1), confirming the overall picture of high similarity from the Mann–Whitney pairwise test. The CVA illustrates a slightly higher morphological divergence between the populations compared to the PCA (Figure 3, Figure S1), though this is expected as the CVA attempts to implement user‐defined groups. The East Usambara population of both females and males (Figure 3) were differentiated from the other populations, likely influenced by the difference in the VSC character as observed in the Mann–Whitney pairwise test, with significant or close to significant values. However, the difference was not significant at the 95% confidence level with CVA‐jack‐knife support values of only 56% and 72% for females and males respectively (Figure 3). Small sample sizes could be affecting the analyses, with several populations represented by fewer than five individuals (SHT, Uluguru and Nguru).

### 3.2 Mitogenome Assembly and Annotation

**FIGURE 4:**
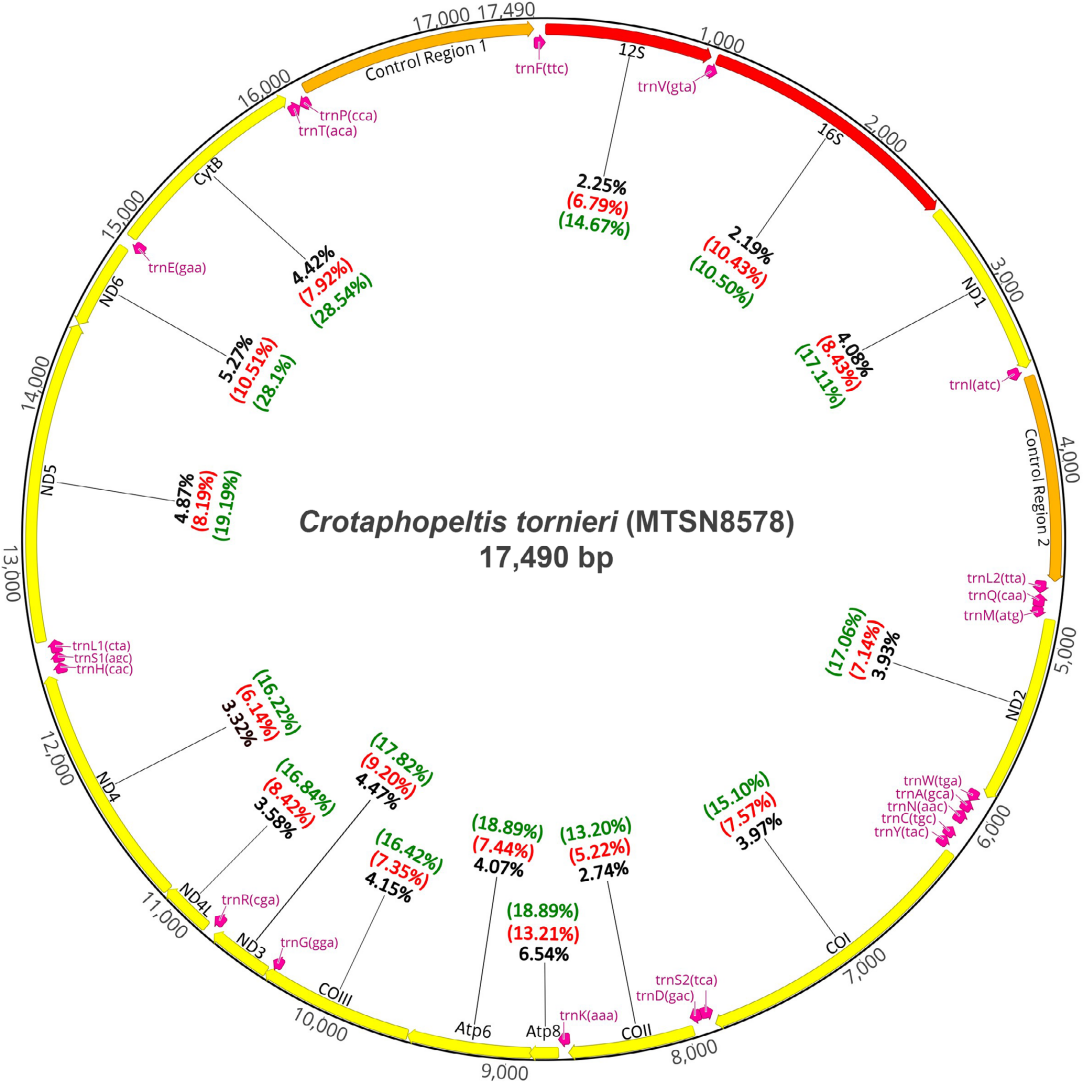
Complete annotated mitogenome of *C. tornieri*; 13 protein‐coding genes (yellow); 2 rRNA (red); control region 1 + 2 (orange); 23 tRNA (pink). Values in black represent the average pairwise genetic distance for each gene between the EAM *C. tornieri* populations. Values in red represent the highest pairwise genetic distance observed between the EAM *C. tornieri* populations. Values in green represent the highest pairwise genetic distance observed between all *C. tornieri* populations, including the SHT populations.

The duplicated Control Region (CR), observed in other colubrid mitogenomes (Yan et al. 2008; Figure 4), proved problematic during the assembly and some positions in these regions could not be determined accurately for all individuals owing partly to low sequence quality in mononucleotide repeat regions, and partly to only a few base pair differences between the two duplicated CR regions. Furthermore, inspections of the mapped BAM‐files revealed that several samples showed signs of polymorphic sites, which should not be the case in the haploid mitogenome. This could either be due to cross‐sample contamination (e.g., while sharing the ethanol solution in the same jar for decades), heteroplasmy, known to occur in snakes (Kumazawa 2004) or possibly the effect of NUMTS showing up in some individuals. Here, we took the conservative approach and simply excluded these samples from the downstream analyses. Despite these obstacles, we were able to assemble complete and high quality mitogenomes from 85 individuals, showing a depth of coverage of 29×–425× with a breadth of coverage of 99.7%–100%. Our dataset of complete mitogenomes included 80 *C. tornieri* (6× SHT, 4× Nguru, 22× West Usambara, 9× East Usambara, 26× Udzungwa, 5× Rubeho, 8× Uluguru, see Figure 1), along with 2× *C. degeni* and 3× *C. hotamboeia* used as outgroups in the phylogenetic analyses. See Table S2 for all sample information.

Based on the highest quality consensus sequence, MTSN8578 (MEA84) with a total of 33.281 mapped reads and 424.8× coverage (Table S2), we annotated the first complete mitogenome of *C. tornieri*. This 17,490 bp sequence consisted of 13 protein coding genes (65.8% of genome), 23 tRNA genes (8.2% of genome), and two ribosomal RNA (13.5% of genome) (Figure 4). The duplicated CRs were spanning from position 3516–4648 (DCR2) and 16,186–17,490 (DCR1) corresponding to 14% of the genome. We found several overlaps between tRNA and protein coding genes, as commonly observed in mitogenomes (Doublet et al. 2015). The mitogenomes of *C. degeni* and *C. hotamboeia* showed the same gene order as *C. tornieri*.

### 3.3 Phylogenetic Analyses

The phylogenetic analyses were based on a 13,964 bp alignment from 87 mitogenomes: 74 *C. tornieri* from across EAM, 6× *C. tornieri* from SHT, two *C. degeni*, three *C. hotamboeia*, one *Lycodon ruhstrati* and one *Gonyosoma frenatum*. The alignment showed 63.3% identical sites and contained 5092 variable sites and 4202 parsimony informative sites, underlining possible significant genetic differences between the individuals. Excluding the outgroups (C*. degeni*, *C. hotamboeia*, *L. ruhstrati* and *G. frenatum*) resulted in 2795 variable sites and 2745 parsimony informative sites, and examining only the EAM populations resulted in 1440 variable sites and 1377 parsimony informative sites (Table 2).

**TABLE 2:** Genetic variability in the sequence alignment rRNA and CDS alignment refers to the full alignment with all three included *Crotaphopeltis* species and two outgroups. *Crotaphopeltis* refers to the alignment with all three included *Crotaphopeltis* species. *C. tornieri* refers to the alignment of only *C. tornieri* individuals. EAM *C. tornieri* refers to the alignment with only *C. tornieri* individuals from the EAM (SHT excluded).

	Alignment Length (bp)	Sequences in alignment	Conserved sites	Segregating sites/variable sites	Parsimony informative sites
rRNA and CDS alignment	13,964	87	63.3%	5092	4202
*Crotaphopeltis*	13,861	85	71.6%	3935	3865
*C. tornieri*	13,772	80	79.6%	2795	2745
EAM *C. tornieri*	13,752	74	89.5%	1440	1377

**FIGURE 5:**
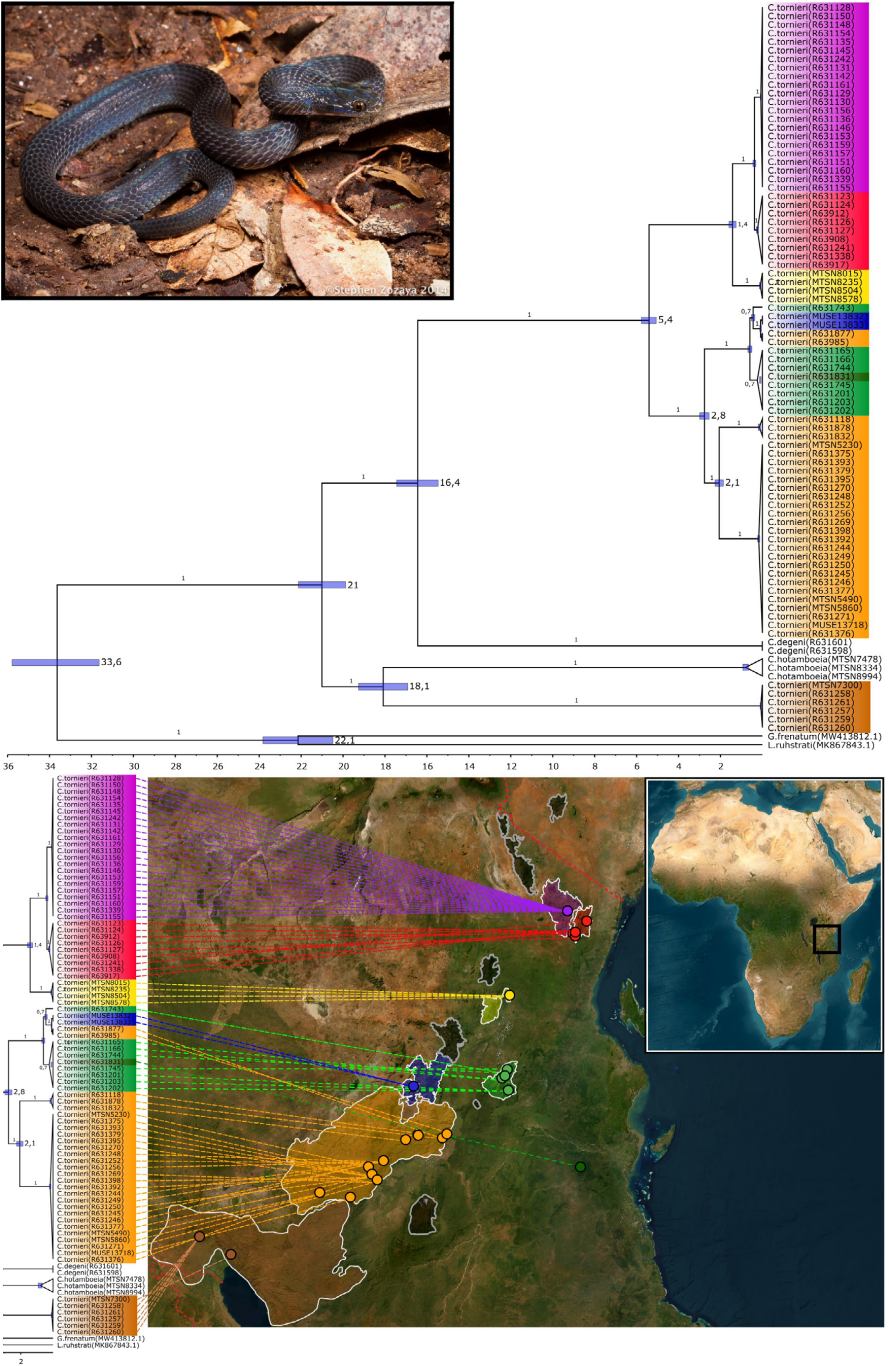
Bayesian Coalescent‐based phylogenetic tree based on CDS and rRNA concatenated alignments. The topology represents the maximum clade credibility tree of 100,000,000 trees and a 10,000,000 tree burn‐in. No pre‐defined outgroup was used. Branch nodes show the posterior probability, and node bars indicate 95% confidence intervals (CI) of the split‐times. The scale bar indicates the time in million years ago (mya) from the present year (0). The species *G. frenatum* and *L. ruhstrati* were used for outgroup purposes, and the phylogenetic tree and coordinate map where conducted using phytools (Revell 2012).

The phylogenetic trees based on Bayesian (Figure 5) and maximum‐likelihood (Figure S2) approaches, along with the haplotype network (Figure S3) showed the same topology. Both trees (Figure 5, Figure S2) reflected a well resolved topology with a 100% node support for most clades and deeply diverged lineages in *C. tornieri* clearly defined by the mountain blocks. Only the Uluguru mountains lineage showed less node support (70% in the BEAST analysis), and only one clade included individuals from several mountain blocks (Rubeho, Uluguru and Northern Udzungwa), showing a wider distribution of this particular mtDNA lineage.

The SHT population rendered *C. tornieri* paraphyletic, and proved highly genetically distant to all other *C. tornieri* clades (Table 3) and was found more similar to *C. hotamboeia* than the EAM *C. tornieri* populations, deviating on average 9.4% and 10%–10.2% for the rRNA and CDS, respectively. The remaining EAM populations were more similar to *C. degeni*, deviating 8.8%–9% on average (Figure 5, Table 3). Assignment of this SHT lineage to *C. tornieri* is therefore probably incorrect, and likely to constitute an undescribed species (see Section 5).

**TABLE 3:** Estimates of Evolutionary Divergence over Sequence Pairs between Groups. The number of base differences per site from averaging over all sequence pairs between groups are shown with the ranges reported in brackets. Standard error estimates are shown above the diagonal. This analysis involved 85 concatenated sequences of 13,964 bp.

Populations	West Usambara	East Usambara	Nguru	Rubeho	Uluguru	Udzungwa	SHT	*C. degeni*	*C. hotamboeia*
West Usambara		0.001	0.004	0.012	0.011	0.012	0.041	0.033	0.041
East Usambara	0.003 (0.002–0.004)		0.003	0.012	0.011	0.012	0.041	0.033	0.041
Nguru	0.012 (0.011–0.013)	0.012 (0.010–0.012)		0.012	0.011	0.012	0.040	0.033	0.041
Rubeho	0.041 (0.034–0.042)	0.040 (0.028–0.042)	0.041 (0.041–0.041)		0.001	0.006	0.039	0.033	0.039
Uluguru	0.040 (0.031–0.042)	0.039 (0.026–0.042)	0.040 (0.038–0.041)	0.005 (0.002–0.005)		0.006	0.039	0.033	0.039
Udzungwa	0.041 (0.034–0.042)	0.041 (0.028–0.042)	0.042 (0.028–0.042)	0.021 (0.001–0.023)	0.021 (0.002–0.023)		0.040	0.034	0.040
SHT	0.121 (0.116–0.122)	0.121 (0.113–0.122)	0.121 (0.120–0.121)	0.117 (0.117–0.117)	0.116 (0.114–0.117)	0.118 (0.117–0.119)		0.039	0.035
*C. degeni*	0.103 (0.98–0.104)	0.103 (0.096–0.104)	0.103 (0.103–0.103)	0.104 (0.104–0.104)	0.102 (0.101–0.104)	0.105 (0.104–0.105)	0.117 (0.117–0.117)		0.040
*C. hotamboeia*	0.121 (0.116–0.122)	0.121 (0.113–0.122)	0.122 (0.122–0.123)	0.117 (0.117–0.118)	0.117 (0.115–0.118)	0.118 (0.117–0.119)	0.110 (0.109–0.110)	0.118 (0.118–0.119)	

Examining the average pairwise distance of rRNA and CDS between the EAM populations, the East Usambara and West Usambara populations were the least diverged, only deviating 0.3%, whereas the Udzungwa and Nguru population showed the highest interpopulation differentiation of 3.6% (Table 3). It is further evident that the EAM populations are structured into a Northern clade (East Usambara, West Usambara and Nguru) and a Southern clade (Udzungwa, Rubeho and Uluguru) (Figure 5), showing a higher genetic differentiation between (3.4%–3.6%) than within the two clades (0.3%–1.8% respectively) (Table 3), supporting the tree topology. Despite observing a clear geographically defined topology with distinct Northern and Southern lineages, the correlation between genetic and geographical distance was insignificant—although only marginally (mantel test, *p* = 0.0108). *Crotaphopeltis hotamboeia* and *C. degeni* were observed to have relatively deep split times with their respective ‘*C. tornieri*’ clade, estimated at 16.4 mya (CI: c. 17.5–15.5 mya) and 18.1 mya (CI: c. 19.2–16.9 mya) respectively (Figure 5). The *C. tornieri* EAM populations split c. 5.4 mya (CI: c. 5.8–5 mya) into the Northern and Southern clade. The Northern clade further diverge into the various mountain populations with the Nguru lineage emerging c. 1.5 mya (CI: c. 1.6–1.3 mya) and the East Usambara and West Usambara diverge c. 0.4 mya (CI: c. 0.4–0.3 mya). In the Southern clade the Uzungwa lineage diverge 2.8 mya (CI: c. 3–2.5 mya) and split further into a northern and southern clade 2.1 mya (CI: c. 2.3–1.9 mya). The Rubeho and Uluguru population diverge c. 0.6 mya (CI: c. 0.7–0.5 mya).

Despite a highly geographically structured mitochondrial gene pool the phylogeny revealed three non‐Rubeho individuals placing in the same clade as individuals from Rubeho (Figure 5). According to the ZMUC collection database, the specimen R631743 was collected from the Uluguru Mts and the specimens R63985 and R631877 from Udzungwa Mts. This could reflect mislabelling or the presence of a single more widespread mitochondrial lineage covering several mountains.

## 4 Discussion

In this study we were able to extract high quality DNA and sequence full mitogenomes from liver samples taken from 85 specimens preserved in museum collections for several decades. All specimens were collected between 1974 and 2014 and are assumed to have been preserved in 70% ethanol since the collection date. The exact preservation history of most specimens is unknown, but formalin exposure could explain why 53 libraries did not provide sufficient quality of DNA for mitogenome assembly. Indeed, some specimens collected in the late 1990s, were injected with 4% formalin after capture (P. Gravlund *pers. com*.). Regardless, these results underline the immense genomic research potential in ethanol‐preserved museum collections when applying a shotgun sequencing approach instead of traditional PCR‐based methods to profile short, damaged DNA fragments (McGuire et al. 2018; Nielsen et al. 2022; Ruane 2021; Zacho et al. 2021).

### 4.1 Morphology

The *C. tornieri* populations were found to be very similar across the 13 morphological traits examined and thus the morphology does not reflect the high genetic variation that we observe between the lineages associated with each mountain. Scale characters are typically highly informative for distinguishing snake species—even closely related ones (Di Nicola 2019; Largen and Rasmussen 1993; Spawls et al. 2018) but here very little clustering is observed in the CVA and PCA analysis (Figure 3, Figure S1) and only 21 of 108 pairwise tests (males and females in total) among all mountain populations showed significant differences. Most of these (15 of the 21) were attributed to the Usambara populations, VSC character of the East Usambara and the SCS character of the West Usambara population (Table 1). This is in agreement with previous studies based on smaller data sets (Barbour and Loveridge 1928; Rasmussen 1993). In addition to the pairwise tests, West Usambara was observed to be different from all other populations in the MSR character, having a count of 19 compared to 17 of the remaining populations (Table S1). There is no obvious explanation as to why the two Usambara populations stand out as different, other than phenotypic drift must have been more pronounced here, perhaps breaking the constraints of morphological conservatisms observed for *C. tornieri* elsewhere across the EAM. Intriguingly, this drift has also resulted in the two Usambara mountain populations being different despite only being separated by a narrow valley (Figure 1, Table 3). This result is not mirrored in the mitogenomes where the two Usambara populations have relatively high genetic similarity to several other populations (Nguru, Uluguru) and also to each other (0.6% pairwise difference) (Table 3). Despite being highly genetically distant from all the EAM *C. tornieri* populations (Table 3), we did not observe any morphological differences between the SHT population and the EAM populations, other than the differences in VSC and MSR to East/West Usambara mentioned above and difference in HP compared to West Usambara and Udzungwa, which could be due to the small sample size (Table 1). In summary, our morphological analyses have included more specimens, more populations, and more characters, than used in previous studies (Gravlund 2002; Rasmussen 1993) but we have reached the same conclusion, namely that the *C. tornieri* populations across the Eastern Arc are highly similar in these scale counts. Thus, the morphology alone does support the general notion of highly stratified populations defined by the mountains in the EAM. As discussed below, this observation is in stark contrast to our genetic results.

### 4.2 Genetic Analyses

Our results show that the EAM *C. tornieri* populations have highly diverged mitochondrial genomes 0.3%–4.1% (Table 3, Figure 5). The pairwise distance between EAM populations ranged from 5.2% to 10.5% for individual genes, with ND6 being the highest. The cytB and COI gene, often used in interspecies phylogenetics (Laopichienpong et al. 2016; Menegon et al. 2014), showed interpopulation distances up to 7.9% and 7.6% respectively (Figure 4). A study of 35 species of snakes from multiple genera in Thailand, found the maximum intraspecies pairwise distance for barcodes of cytB and COI to be 2.1% and 11.7% respectively (Laopichienpong et al. 2016), and a study of garter snakes found intraspecies divergence in mtDNA between 2.4% and 6.6% (de Queiroz, Lawson, and Lemos‐Espinal 2002). Furthermore, the pairwise differences between congeneric species has been shown to be as low as 3.2%–5.6% and 4.2% based on concatenated sequences of cytB and ND4 (Atheris, Menegon et al. 2014) and COI and Cytb (Laopichienpong et al. 2016), respectively. The mtDNA lineages of the EAM *C. tornieri* populations are indeed as diverged as observed between many recognised species implying that cryptic speciation is a likely scenario.

The phylogenetic tree shows a highly structured mitochondrial gene pool in *C. tornieri* defined by the mountains, with deep matrilineal splits and very high node support values (Figure 5). This is also observed directly in the high genetic distances between populations showing that they have been isolated long enough for genetic drift to significantly affect the gene pools (Table 3). Thus, our genetic results conform with the pattern found in several genetic studies on the fauna of the EAM (e.g., Beresford, Fjeldså, and Kiure 2004; Dimitrov, Nogues‐Bravo, and Scharff 2012; Tolley et al. 2011; Menegon et al. 2014; Nielsen et al. 2022), including previous studies of *Crotaphopeltis* (Gravlund 2002), namely that the Eastern Arc Mountains harbour distinct genetic units both at species and population level. The first observed split within the EAM *C. tornieri* occurs c. 5.4 mya (CI: c. 5.8–5 mya) (Figure 5), with the lineage splitting into a Northern and Southern clade (Table 3, Figure 5), a trend also observed in other species (Blackburn and Measey 2009; Tolley et al. 2011). The split into the two major clades is also apparent in the pairwise genetic distance, showing higher similarity between populations within the Northern and the Southern clade, respectively (Table 3). This could imply the presence of a panmictic population prior to this north–south split, likely occupying a larger coherent rainforest in a less arid environment (Howell 1993; Lovett 1993a, 1993b; Wasser and Lovett 1993).

Aridification might have happened during this time, as the uplifting of the current EAM blocks 7–2 mya (Griffiths 1993; Ring 2014; Lovett and Wasser 2008), altered the climate of the region, causing the rainforest to retract and savannah becoming more prominent (Ségalen, Lee‐Thorp, and Cerling 2007; Sepulchre et al. 2006). The phylogenetic topology suggests that the forest initially broke up somewhere between Rubeho (Southern clade) and Nguru (Northern clade) (Figure 5), resulting in a Northern and a Southern Forest fragment separated by savannah. The more distal phylogenetic splits indicate that the forest continued to retract towards the uplifting mountains, where humid oceanic wind condenses and ensures precipitation (Lovett 1993a, 1993b; Wasser and Lovett 1993). Because the oldest observed split within *C. tornieri* was between the Northern and Southern clade, we decided to further examine for morphological differences between the Northern and Southern populations. The Population from the Northern and Southern regions were grouped and compared in the Mann–Whitney pairwise test. We found that the Northern and Southern population are significantly different in the VSC for both males and females, and in the SCS for the females, consistent with the results shown in Table 1. *Crotaphopeltis tornieri* further split into isolated lineages, likely following a retracting forest (c. 2.8 mya Southern clade and c. 1.4 mya Northern clade) (Figure 5). A cooler and drier climate occurred at this time 3–2 mya, as the Arctic started to freeze, presumably causing the rainforest to retract further (Polyak et al. 2010).

The Northern clade splits into well‐defined geographically distinct lineages on each mountain (Figure 5) and the Southern clade split into distinct lineages as well, albeit with some exceptions as the mtDNA lineage found on Rubeho is also present at both Uluguru and Udzungwa, and the single Kichi Hill individual is similar to the Uluguru mtDNA lineage (Figure 5). These few widespread mitochondrial lineages could reflect recent dispersal or traces of a former connected gene pool, or they could also be due to locality mislabelling, which is difficult to completely rule out in this case where some specimens have been brought by locals. Especially the Rubeho and Udzungwa mountains are in relatively close proximity and have elevated rocky terrain between them. An elevated rocky passage is also present between Udzungwa and Uluguru, though the distance here is significantly longer (> 50 km). Thus, a recent dispersal seems more unlikely here. Furthermore, the Mantel test showed no significant correlation (*p* = 0.108) between the genetic distance and geographical distance between the mountains, which would be expected if the gene pools on the mountains were connected. An additional mantel test was conducted without these widespread clades but this did still not result in a significant correlation between genetic and geographic distance (*p* = 0.129). We further observe that the Udzungwa population is split into a Northern and Southern subpopulation, with a relatively deep split and another clade (with individuals from Northern Udzungwa) that is less geographically well‐defined.

(Figure 5). This finding could indicate that biogeographically distinct units have formed within the Udzungwa mountain block, perhaps in this case supporting the ‘elevated in situ speciation rate’ hypothesis (Burgess et al. 2007; Fjeldsaå and Lovett 1997) as one of the evolutionary mechanisms involved in Udzungwa. The highly distinct clade structure and high levels of differentiation rejects the scenario of many recent dispersals populating the mountains. Thus, the highly conserved morphology within the species, seems best explained by fracturing of an old widespread lineage, supporting the ‘species pump’ hypothesis, especially when combined with phylogenetic niche conservatism (Shepard and Burbrink 2009; Wiens 2004). *Crotaphopeltis tornieri* likely resembles the morphology of an ancestral forest species, possibly occupying the same 30 million old forests they live in today (Wasser and Lovett 1993), resulting in morphological conservatism between highly genetically diverged populations (Table 3). This is supported by the phylogenetic topology presented in this study, with two deep paraphyletic lineages with no difference in the examined morphologcal characters (Figure 5).

### 4.3 A Highly Distinct Lineage at SHT

Our results have confirmed that specimens assigned to *C. tornieri* are genetically paraphyletic (in agreement with Gravlund (2002)) on the whole mitogenome level, in respect to both *C. hotamboeia* and also *C. degeni*. The SHT *C. tornieri* population is almost equally diverged from *C. hotamboeia*, *C. degeni* and the EAM *C. t* *orni* *er* *i* (Table 3), indicating that the SHT population became isolated before the EAM populations diverged, and the estimated split time is indeed very old; 21 mya (Figure 5).

It would be tempting to leverage the SHT lineage to a separate cryptic species with potentially phylogenetic niche conservatism with *C. tornieri* (Wiens 2004; Wiens and Graham 2005). The pairwise distances clearly show that the SHT populations are highly different from the EAM populations (Table 3). The individual gene regions showed pairwise distances up to 10.5%–28.5% with cytB being the highest (Figure 4). With the average pairwise distance for cytB in reptiles being 13.6%, measured within 35 genera (Harris 2002), the mtDNA divergence between EAM and SHT populations are extremely high for the same presumed species. Phylogenetic studies of snakes in the EAM found interspecies pairwise distance based on 1737 bp sequences of cytB and ND4 to be 3.2%–14.8% (Menegon et al. 2014), which is much lower compared the EAM and SHT *C. tornieri* populations showing an average divergence of 15.8% (ND4) and 27% (cytB). Furthermore, a study of the COI gene in snakes of a comparable island structure in the Socotra Archipelagos, found intraspecific pairwise distance (< 9%) to be similar to the highest observed intrapopulation distance of EAM populations (7.6%) and the interspecific divergence (> 14%) to be similar to the EAM and SHT *C.* *torni* *e* *r* *i* populations (13.7%–15.1%) (Vasconcelos et al. 2016). The critera for determining species boundaries based on genetic data are not clear cut (Padial et al. 2010). Yet strongly supported phylogenetic placements (Laopichienpong et al. 2016; de Queiroz, Lawson, and Lemos‐Espinal 2002) and divergence estimates above 9% has been used to recognise candidate species (Laopichienpong et al. 2016; Vasconcelos et al. 2016). Clearly, the SHT and EAM lineages in *C. tornieri* present as a strong candidates for being different species and conducting thorough morphological analyses of characters not examined in this study, for example, skeletal structures, could be highly informative. The SHT and EAM populations could represent cryptic species according to typical mtDNA cutoffs (Fišer, Robinson, and Malard 2018; Laopichienpong et al. 2016; Vasconcelos et al. 2016); although we cannot here completely rule out mitonuclear discordance via either introgression or incomplete lineage sorting (Thanou et al. 2020); or systematic errors in shape of long branch attraction (Bergsten 2005). However, to investigate those scenarios further, additional research needs to be concluded on the nuclear genome level, along with a more comprehensive sampling in the SHT.

### 4.4 Concluding Remarks

Analyses found very high levels of genetic differentiation between the EAM *C. tornieri* populations. This supports the theory of long isolation periods due to vicariance events, and this was corroborated by seeing a possible correlation between split times and past climate changing events during the uplifting of the EAM. However, we also found indications for more recent diversification within the Udzungwa Mountain, perhaps supporting the ‘elevated in situ speciation’ theory. We found high morphological conservatism in *C. tornieri*, indicative of a highly preserved ancestral morphology, perhaps adapted to rainforest. The SHT population was found to be highly genetically distinct despite having a similar morphology, possibly being a cryptic species. However, in order to unravel the full evolutionary history of this genus, we would ideally have all *Crotaphopeltis* species represented in the phylogeny, and additional nuclear genome wide data allowing for more complex demographic analyses (e.g., Gutenkunst et al. 2009; Li and Durbin 2011; Korneliussen, Albrechtsen, and Nielsen 2014).

## Author Contributions

**Tejs L. Nielsen:** data curation (lead), formal analysis (lead), investigation (lead), methodology (supporting), project administration (equal), supervision (equal), visualization (lead), writing – original draft (lead), writing – review and editing (lead). **Sofie Holdflod Nielsen:** data curation (equal), formal analysis (supporting), investigation (equal), methodology (supporting), project administration (supporting), validation (supporting), visualization (supporting), writing – original draft (supporting). **Maria Novosolov:** formal analysis (supporting), methodology (supporting), supervision (supporting), visualization (supporting), writing – original draft (supporting). **Peter Gravlund:** conceptualization (supporting), investigation (supporting), supervision (supporting), writing – original draft (supporting). **Morten E. Allentoft:** conceptualization (lead), formal analysis (supporting), funding acquisition (lead), investigation (supporting), methodology (lead), project administration (equal), resources (equal), supervision (lead), validation (equal), visualization (supporting), writing – original draft (supporting), writing – review and editing (supporting).

## Conflicts of Interest

The authors declare no conflicts of interest.

## Supporting information


**Figure S1.** PCA‐plots of morphological data of 103 females (A) and 78 males (B) including all traits examined besides ‘KVS’.

**Figure S2.** Maximum‐likelihood‐based phylogenetic tree build in RAxML‐NG v. 0.8.1 Beta based on CDS and rRNA concatenated alignments.

**Figure S3.** Haplotype network using a minimum spanning network in Popart (Bandelt, Forster, and Röhl 1999), only including *Crotaphopeltis* species in the alignment.

**Table S1.** Morphometric data summarising the morphological results for each *C. tornieri* population.

**Table S2.** Assembly information regarding the 85 mitogenomes generated and used in the phylogenetic analyses.

## Data Availability

The 85 mitochondrial genomes sequenced and assembled in this study have been deposited in GenBank with accession numbers as follows: East Usambara (PV088961‐PV088969), West Usambara (PV068222‐PV068243), Nguru (PV013520‐PV013523), Rubeho (PV068158‐PV068159), SHT (PV126274‐PV126279), Udzungwa (PV068244‐PV068266), Udzungwa, North clade (PV068157, PV068160, PV068097‐PV068099), Uluguru (PV068161, PV031957‐PV031963), Kichi Hill, Uluguru clade (PV031964), *C. degeni* (PQ778320‐PQ778321), *C. hotamboeia* (PV068163‐PV068165).
