# Supplementary figures and images for: Deeply Diverged but Morphologically Conserved Lineages in Tornier's Cat Snake (Crotaphopeltis tornieri) of the Eastern Arc Mountains
Source: Ecol Evol. 2025 Feb 25;15(2):e70452. doi: 10.1002/ece3.70452 (PMC11850987; doi:10.1002/ece3.70452)

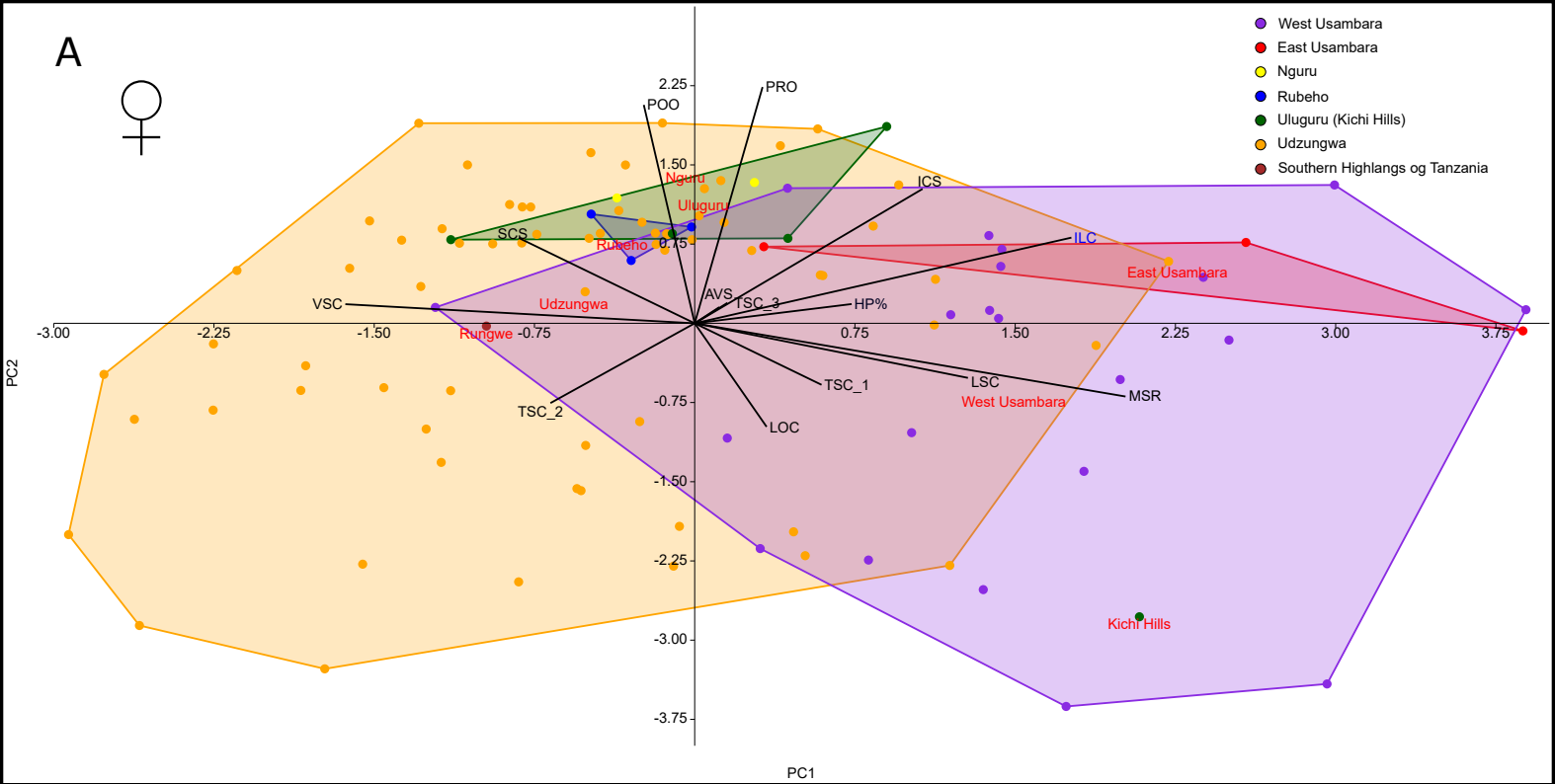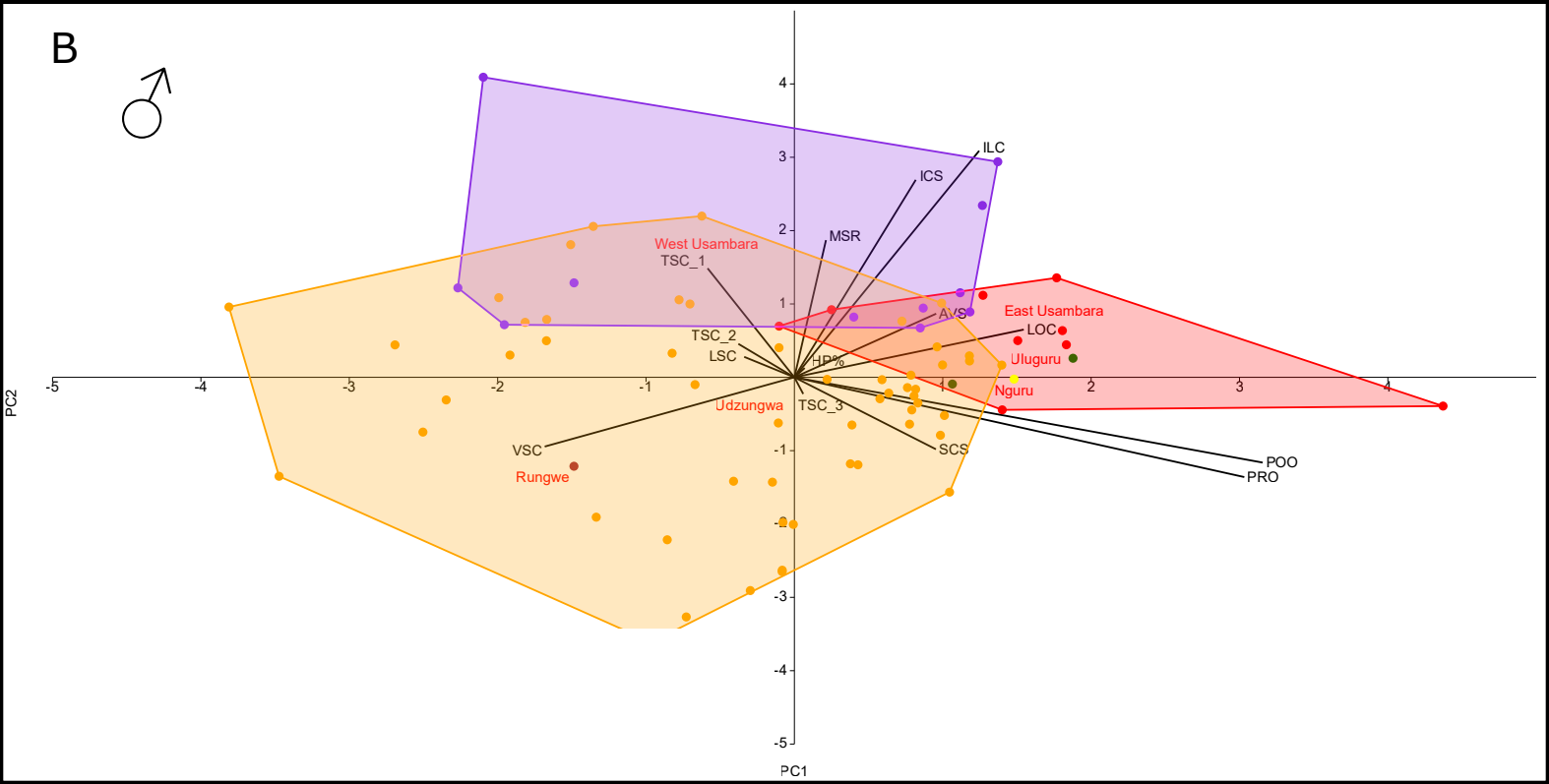

Supplement: Supplementary file 1 — Figure S1. PCA‐plots of morphological data of 103 females (A) and 78 males (B) including all traits examined besides ‘KVS’. [file ECE3-15-e70452-s001.pdf]
