# Supplementary material for: Deeply Diverged but Morphologically Conserved Lineages in Tornier's Cat Snake (Crotaphopeltis tornieri) of the Eastern Arc Mountains
Source: Ecol Evol. 2025 Feb 25;15(2):e70452. doi: 10.1002/ece3.70452 (PMC11850987; doi:10.1002/ece3.70452)
Supplement: Supplementary file 4 — Table S1. Morphometric data summarising the morphological results for each C. tornieri population. [file ECE3-15-e70452-s003.docx]

|  | **VSC** | **AVS** | **SCS** | **HP** | **MSR** | **TSC** | **LSC** | **LOC** | **ILC** | **ICS** | **PRO** | **POO** | **KVS** |
| --- | --- | --- | --- | --- | --- | --- | --- | --- | --- | --- | --- | --- | --- |
| **West Usambara** | | | | | | | | | | | | | |
| ♂  **(13)** | 157-171  (13) | 0-2  (13) | 37-48  (13) | 19,3-22,2% (11) | 17 (2)  19 (11) | 1+2 (10)  2+2 (1)  1+1+2 (1)  1+2+2(1) | 8 (12)  9 (1) | 2 (1)  3 (12 | 9 (3)  10 (7)  11 (3) | 4 (1)  5 (11)  6 (1) | 1 (5)  2 (8) | 1 (5)  2 (8) | Yes  (13) |
| ♀  (**21)** | 161-172  (21) | 0-2  (21) | 32-48  (21) | 16,1-23,6% (21) | 19 (18)  17 (3) | 1+2 (8)  2+2 (1)  1+1+2 (6)  1+2+1 (4)  1+2+2 (1)  2+2+2 (1) | 7(1)  9 (7)  8 (13) | 2 (1)  3 (20) | 11 (1)  9 (2)  10 (18) | 4 (2)  5 (19) | 1 (6)  2 (15) | 1 (6)  2 (15) | Yes (21) |
| **East Usambara** | | | | | | | | | | | | | |
| ♂  **(11)** | 147-156  (10) | 0-2  (11) | 43-49  (11) | 18,9-23,3% (10) | 17 (10)  19 (1) | 1+2 (9)  1+1+2 (1)  1+2+2 (1) | 8 (10)  9 (1) | 3 (11) | 9 (1)  10 (9)  11 (1) | 5 (11) | 1 (3)  2 (6)  3 (1) | 1 (1)  2 (9)  3 (1) | Yes  (11) |
| ♀  **(5)** | 146-161  (4) | 0-2  (5) | 42-48  (5) | 18,8-24%  (3) | 17 (1)  19 (4) | 1+2 (3)  1+1+2 (2) | 8 (3)  9 (2) | 3 (5) | 10 (4)  11 (1) | 5 (4)  6 (1) | 2 (5) | 2 (5) | Yes (4)  No (1) |
| **Nguru** | |  |  |  |  |  |  |  |  |  |  |  |  |
| ♂  **(2)** | 163-166  (2) | 1-2  (2) | 46-48  (2) | 19,6-23,5% (2) | 17 (2) | 1+2 (1)  1+1+2 (1) | 8 (2) | 3 (2) | 10 (1) | 5 (1) | 2 (2) | 2 (2) | Yes  (2) |
| ♀  (**2)** | 161-169  (2) | 0-1  (2) | 39-41  (2) | 18,9-20,5% (2) | 17 (2) | 1+2 (2) | 8 (2) | 2 (2) | 10 (2) | 5 (2) | 2 (2) | 2 (2) | Yes  (2) |
| **Uluguru** | |  |  |  |  |  |  |  |  |  |  |  |  |
| ♂  **(3)** | 154-164  (2) | 1-2  (2) | 49-52  (2) | 20,7-21,4% (2) | 17 (2) | 1+2  (3) | 8 (3) | 3 (3) | 10 (3) | 5 (3) | 2 (3) | 2 (3) | Yes  (3) |
| ♀  **(4)** | 160-168  (4) | 1-2  (4) | 43-50  (4) | 19,4-22,6% (4) | 17 (4) | 1+2 (4) | 8 (4) | 3 (4) | 9 (3)  10 (1) | 5 (3)  6 (1) | 2 (4) | 2 (4) | Yes  (4) |
| **Rubeho** | |  |  |  |  |  |  |  |  |  |  |  |  |
| ♀  **(3)** | 167-179  (3) | 0-2  (3) | 41-44  (3) | 18,3-24% (3) | 17 (3) | 1+2 (3) | 8 (3) | 3 (3) | 10 (3) | 5 (3) | 2 (3) | 2 (3) | Yes  (3) |
| **Udzungwa** | |  |  |  |  |  |  |  |  |  |  |  |  |
| ♂  **(66)** | 155-178  (61) | 0-3  (64) | 29-53  (63) | 15,5-22,8%  (59) | 17 (59)  19 (7) | 1+2 (51)  1+3(1)  2+2 (1)  1+1+2 (5)  1+2+1 (2)  1+2+2 (3)  2+2+2 (1) | 7 (1)  8 (59)  9 (3) | 2 (7)  3 (57) | 8 (7)  9 (17)  10 (37) | 4 (14)  5(46)  6 (1) | 1 (21)  2 (44)  3 (1) | 1 (15)  2 (51) | Yes  (65)  No  (1) |
| ♀  **(83)** | 161-187  (77) | 0-2  (83) | 32-49  (80) | 15,3-22,9%  (74) | 17 (73)  18 (1)  19 (9) | 1+1 (1)  1+2 (55)  1+3 (1)  2+2 (2)  1+1+1(2)  1+1+2 (6)  1+1+3 (1)  1+2+1 (4)  1+2+2 (7)  2+1+2 (1) | 7 (1)  8 (69)  9 (8) | 2 (13)  3 (66) | 7 (1)  8 (6)  9 (23)  10 (47) | 4 (14)  5 (59)  6 (4) | 1 (23)  2 (57) | 1 (16)  2 (62)  3 (1) | Yes  (79)  No (4) |
| **SHT** | |  |  |  |  |  |  |  |  |  |  |  |  |
| ♂  **(2)** | 165-167  (2) | 1 (2) | 45-46  (2) | 21,2-21,6%  (2) | 17 (1) | 1+2 (1)  1+1 (1) | 9 (1)  8 (1) | 3 (2) | 9 (1)  10 (1) | 4 (1)  5 (1) | 2 (2) | 1 (1)  2 (1) | Yes  (1)  No (1) |
| ♀  **(2)** | 166-169  (2) | 0-1  (2) | 35-41  (2) | 22,5-22,9% (2) | 17 (1)  18 (1) | 1+2 (1)  1+2+2 (1) | 8 (1)  9 (1) | 2 (1)  3 (1) | 9 (2) | 4 (2) | 2 (2) | 2 (1) | Yes (2) |
| Kichi Hills | | | | | | | | | | | | | |
| ♀  **(1)** | 156 (1) | 1 (1) | 35 (1) | 18,6% (1) | 19 (1) | 1+2 (1) | 8 (1) | 8 (1) | 10 (1) | 5 (1) | 1 (1) | 1 (1) | Yes (1) |
