# Supplementary material for: Deeply Diverged but Morphologically Conserved Lineages in Tornier's Cat Snake (Crotaphopeltis tornieri) of the Eastern Arc Mountains
Source: Ecol Evol. 2025 Feb 25;15(2):e70452. doi: 10.1002/ece3.70452 (PMC11850987; doi:10.1002/ece3.70452)
Supplement: Supplementary file 2 — Figure S2. Maximum‐likelihood‐based phylogenetic tree build in RAxML‐NG v. 0.8.1 Beta based on CDS and rRNA concatenated alignments. [file ECE3-15-e70452-s005.pdf]

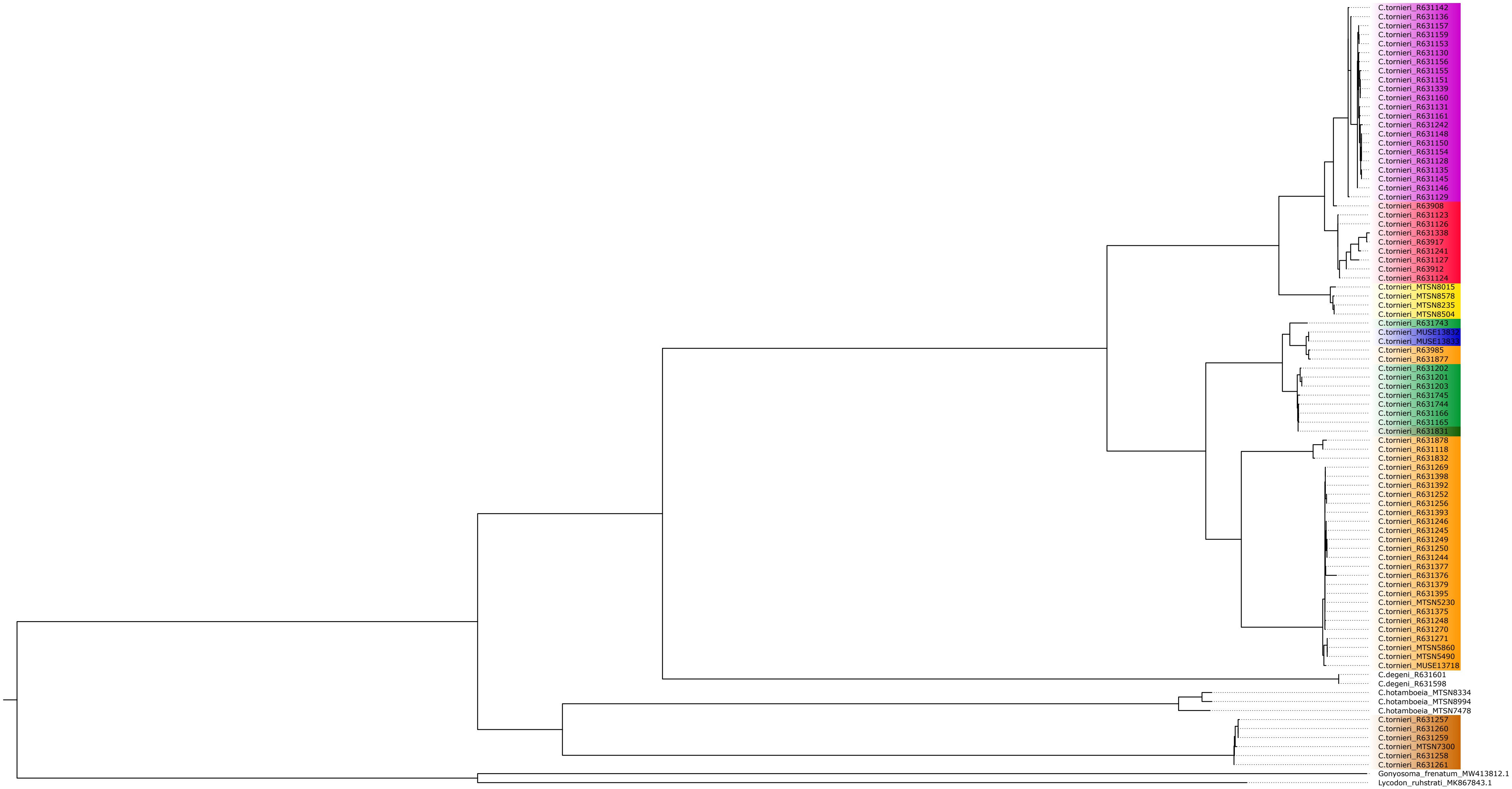
C.tornieri_R631142
C.tornieri_R631136
C.tornieri_R631157
C.tornieri_R631159
C.tornieri_R631153
C.tornieri_R631130
C.tornieri_R631156
C.tornieri_R631155
C.tornieri_R631151
C.tornieri_R631339
C.tornieri_R631160
C.tornieri_R631131
C.tornieri_R631161
C.tornieri_R631242
C.tornieri_R631148
C.tornieri_R631150
C.tornieri_R631154
C.tornieri_R631128
C.tornieri_R631135
C.tornieri_R631145
C.tornieri_R631146
C.tornieri_R631129
C.tornieri_R63908
C.tornieri_R631123
C.tornieri_R631126
C.tornieri_R631338
C.tornieri_R63917
C.tornieri_R631241
C.tornieri_R631127
C.tornieri_R63912
C.tornieri_R631124
C.tornieri_MTSN8015
C.tornieri_MTSN8578
C.tornieri_MTSN8235
C.tornieri_MTSN8504
C.tornieri_R631743
C.tornieri_MUSE13832
C.tornieri_MUSE13833
C.tornieri_R63985
C.tornieri_R631877
C.tornieri_R631202
C.tornieri_R631201
C.tornieri_R631203
C.tornieri_R631745
C.tornieri_R631744
C.tornieri_R631166
C.tornieri_R631165
C.tornieri_R631831
C.tornieri_R631878
C.tornieri_R631118
C.tornieri_R631832
C.tornieri_R631269
C.tornieri_R631398
C.tornieri_R631392
C.tornieri_R631252
C.tornieri_R631256
C.tornieri_R631393
C.tornieri_R631246
C.tornieri_R631245
C.tornieri_R631249
C.tornieri_R631250
C.tornieri_R631244
C.tornieri_R631377
C.tornieri_R631376
C.tornieri_R631379
C.tornieri_R631395
C.tornieri_MTSN5230
C.tornieri_R631375
C.tornieri_R631248
C.tornieri_R631270
C.tornieri_R631271
C.tornieri_MTSN5860
C.tornieri_MTSN5490
C.tornieri_MUSE13718
C.degeni_R631601
C.degeni_R631598
C.hotamboeia_MTSN8334
C.hotamboeia_MTSN8994
C.hotamboeia_MTSN7478
C.tornieri_R631257
C.tornieri_R631260
C.tornieri_R631259
C.tornieri_MTSN7300
C.tornieri_R631258
C.tornieri_R631261
Gonyosoma_frenatum_MW413812.1
Lycodon_ruhstrati_MK867843.1
